# EGFR Mutations and Lung Cancer

**DOI:** 10.1371/journal.pmed.0020374

**Published:** 2005-10-04

Tyrosine kinases of the epidermal growth factor receptor (EGFR) family are frequently mutated in human cancers. Mutations in the tyrosine kinase domain of EGFR (encoded by exons 18–24) have mostly been found in lung cancers. Some, but not all, lung cancers carrying such mutations are responsive to treatment with small-molecule EGFR inhibitors, including the two FDA-approved drugs erlotinib and gefitinib.

Matthew Meyerson and colleagues undertook a systematic study of the different classes of EGFR mutations found in lung cancers in order to understand their roles in tumorigenesis on one hand, and their relation to drug sensitivity on the other. They introduced different altered EGFR versions into fibroblast and lung epithelial cells, and found that all of the mutant proteins transformed both cell types in an EGF-independent manner. Transformation was associated with constitutive kinase activity and with the activation of known downstream signaling pathways.

While the various mutant receptors had similar transforming capabilities, cells expressing them differed in their response to EGFR inhibitors. Transformation of cells expressing mutations in exons 18, 19, and 21 was inhibited by 100 nM erlotinib or gefitinib, whereas no significant inhibitory effect on cells expressing an exon 20 insertion mutation was seen even at much higher concentrations of either drug. This result is consistent with the lack of clinical responses to erlotinib or gefitinib in three lung cancer patients with exon 20 mutations. In contrast, when the researchers tested another experimental EGFR inhibitor called CL-387,785, they found cells expressing the exon 20 insertion mutation to be sensitive, consistent with previous studies that had found similar patterns with other EGFR exon 20 mutations. [Fig pmed-0020374-g001]

**Figure pmed-0020374-g001:**
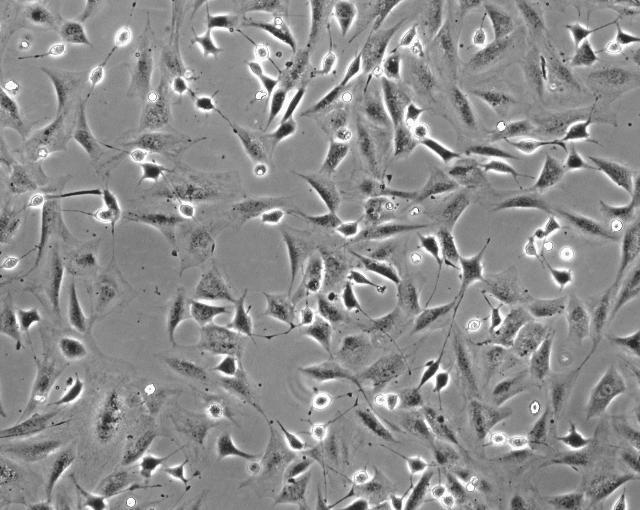
Morphology of fibroblasts expressing mutant EGFR

These results highlight the problems and the possibilities of individualized cancer therapy. One drug is unlikely to fit all tumors, not even all tumors with mutations in a specific oncogene. On the other hand, having a collection of drugs against a particular target increases chances that one of them will prove effective, and that alternatives exist when tumors develop resistance. Developing such a collection and selecting the right drug for the right patient is a challenge not only scientifically but also economically.

